# The Progression of Nearwork Myopia

**DOI:** 10.4172/2476-2075.1000120

**Published:** 2016-07-20

**Authors:** Peter R Greene, Antonio Medina

**Affiliations:** 1 Bioengineering, BGKT Consulting Ltd, Huntington, New York, USA; 2 Research, MultiVision, Milpitas, CA, USA

**Keywords:** Feedback, Emmetropization, Myopia, Reading glasses

## Letter to the Editor

The purpose of this letter is to present a simplified mathematical model of progressive myopia. A proposed hypothesis for refractive error development of the human eye requires that there is an optical signal related to the amount of refractive error which would in turn correct the refractive error of the eye. A specific first-order feedback system, defined by the transfer function F (s) = 1 / (1+ks) [1] was proposed by Medina and Fariza in 1993 [1].

This particular feedback control is termed here “Feedback Theory.” This Feedback Theory for emmetropization was based on and supported by data from children and young adults and as such it is applicable to at least those ages [1]. This Feedback Theory is a quantitatively mathematical theory that explains how the refraction of the eye changes over time and the effect of ophthalmic lenses. Feedback Theory explains what happens, rather than how it happens. According to Feedback Theory the monitored signal that is fed back is the refractive state R (t) [diopters], not the information on accommodation state or other optical sources.

Nearwork has been proposed as a factor in myopia progression [2]. Near work, such as intense computer work, increases myopia [3,4]. We argue that the Feedback Theory also predicts causative relationship between nearwork and myopia as nearwork is equivalent to added minus (negative) lenses while looking in the distance. The nearwork dioptric value, or the dioptric value of the equivalent lens can be added as a step input to the feedback system [5], which would then respond increasing the amount of myopia.

Some have argued that the Feedback Theory is defined in the space domain instead of the time domain and have therefore been disinclined to work with Feedback Theory. However, we show here how the response of a first-order feedback system in the time domain is a decaying exponential, as seen in many physical and physiological phenomena, and it describes a change in myopia with time.

The system output in the t-domain is the inverse Laplace transform of the output in the s-domain, which in turn is the transfer function times the step input in the s-domain:

Eq. (1) $$\begin{array}{cc} & o(t)=L^{-1}\{O(s)\}=L^{-1}\{I(s)F(s)\}=L^{-1}\{L[i(t)]F(s)\}=\\ & L^{-1}\{-3∕[s(1+\mathrm{ks})]\}=-3+3\;\text{exp}\;(-t∕k)=-3[1-\text{exp}\;(-t∕k)] \end{array}$$

where t is time, k is the time constant RC, I(s) is the step input in the s-domain, i(t) is the step input in the time domain (−3 D in our example), O(s) is the output in the s-domain and o(t) is the output in the time domain, L is the Laplace transform and L^−1^ is the inverse Laplace transform.

**Figure 1:**
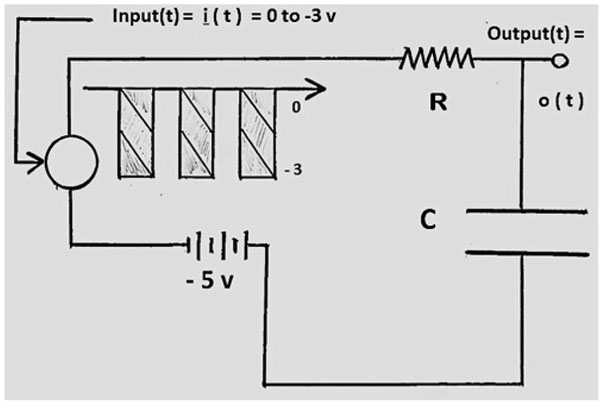
Electrical circuit shows the refractive development as the voltage at the capacitor of a corrected −5 D eye, periodically reading at a distance of 1/3 meter (14 inches).

An electric circuit for example can simulate myopia progression vs. time R(t) because the response of the feedback system to a lens step input is the same as the capacitor voltage in a R-C (Resistor-Capacitor) circuit, as shown in Figure 1. When near work is involved a negative square-wave represents the negative lens equivalent to the accommodative demand, as represented in the inset Figure 1.

In order to numerically solve, this is a fairly complex calculation. The R-C circuit solves the problem without any computations, Figure 1. The system exhibits an exponential progression of the capacitor voltage o(t) [or refraction R(t)] [1] [6],

Eq. (2) R(t)=−5.0D−3.0D[1−exp(−t∕k)]

where t is time, k is the time constant and R is either refraction or voltage. This equation applies initially when the square wave is at −3, and then exponentials alternating with the square wave apply as described in [6]. This electrical circuit simulates myopia progression vs. time as the voltage at the capacitor, where Volts (V) represent Diopters (D) and a negative square-wave represents the effect of intermittent near work, equivalent to the use of a negative lens.

The use of this analogy illustrates how near work triggers a myopizing progression additional to that created by the negative lens applied to the eye to correct myopia. This analog-circuit technique is a general result, which as a practical matter, can accommodate many different variations of parameters and initial conditions. For instance, the system response to under-correction [7] is given by simply replacing the −5 v battery with a lower voltage battery.
